# Supplementary figures and images for: Temporal changes in spike IgG levels after two doses of BNT162b2 vaccine in Japanese healthcare workers: Do spike IgG levels at 3 months predict levels 6 or 8 months after vaccination?
Source: PLoS One. 2022 Jun 17;17(6):e0263486. doi: 10.1371/journal.pone.0263486 (PMC9205520; doi:10.1371/journal.pone.0263486)

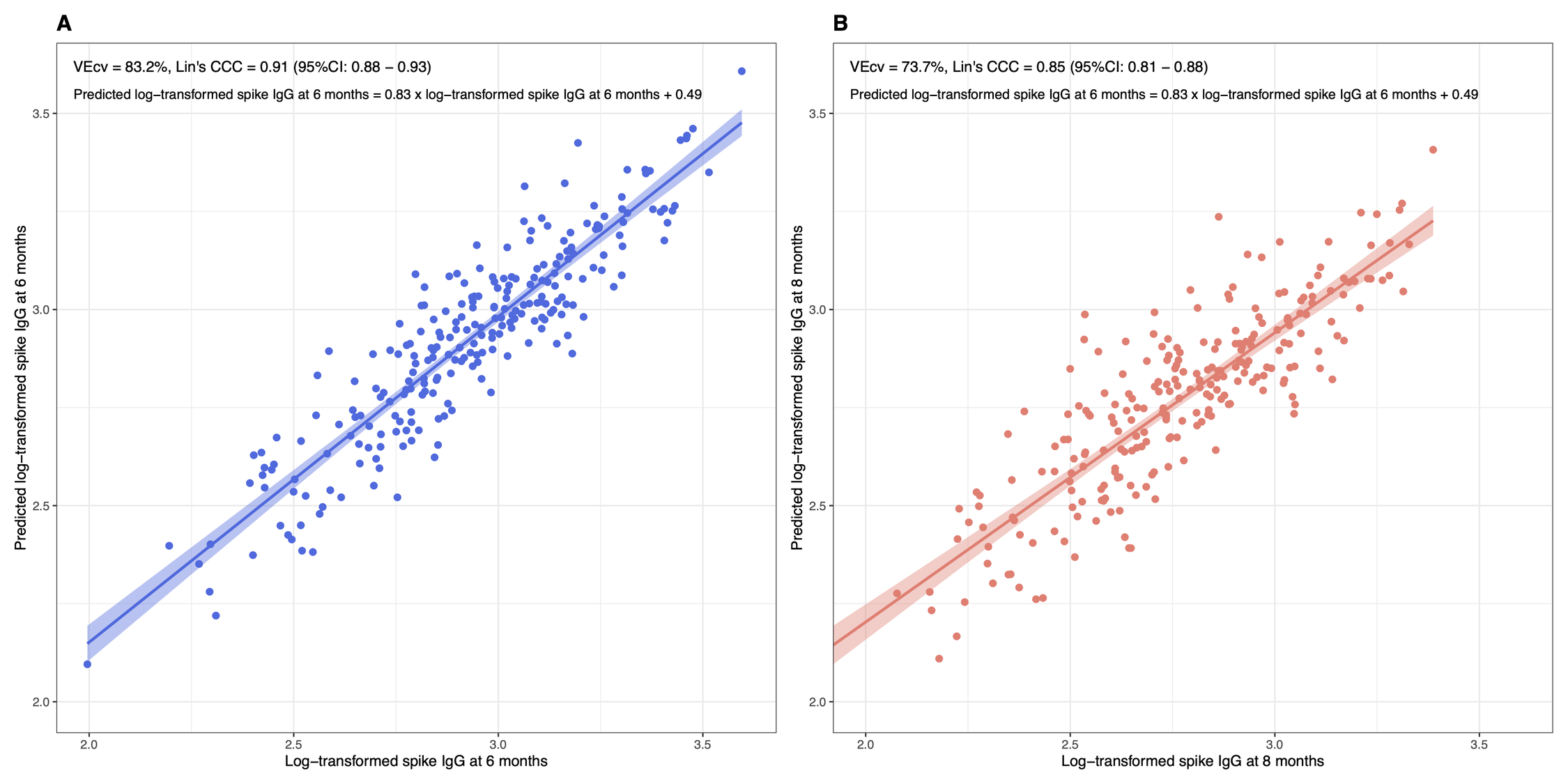

Supplement: S1 Fig — A linear correlation between observed spike IgG level (x-axis) and predicted spike IgG level (y-axis) at 6 months (panel A), at 8 months (panel B). (TIF) [file pone.0263486.s001.tif]
