# Supplementary material for: Temporal changes in spike IgG levels after two doses of BNT162b2 vaccine in Japanese healthcare workers: Do spike IgG levels at 3 months predict levels 6 or 8 months after vaccination?
Source: PLoS One. 2022 Jun 17;17(6):e0263486. doi: 10.1371/journal.pone.0263486 (PMC9205520; doi:10.1371/journal.pone.0263486)
Supplement: Attachment — Submitted filename: PLOS One COVID-19 Vaccine Comments to Authors-1.docx [file pone.0263486.s003.docx]

**Comments for the authors**

The study conducted by Takeuchi et al., entitled " Prediction of Spike IgG Levels in Japanese Workers 6 and 8 months after the Second Dose of BNT162b2 Vaccine" was designed to detect the levels of anti-S IgG at 3, 6, and 8 months after two doses of BNT162b2 Vaccine. The authors hypothesized that levels of anti-S IgG at three months after two doses predict the levels after 6 and 8 months based on which one can determine if a third dose is needed within a fixed time frame.

This study is an extension of the previous study by the authors where they hypothesized that the reactogenicity after two doses of the BNT162b2 vaccine may predict spike IgG antibody levels.

Results of this study are consistent with current literature in the field. However, on the whole, the paper is not suitable for publication in its present form.

**Specific Comments:**

At the time of the study, booster (3^rd^ dose) was already approved and in use in many countries. It is now common globally. Hence studying IgG levels at 8 months after the second dose make the significance of this study questionable. Also, it is important to perform measurements of neutralizing antibody titers, which are a better reflection of protective immune responses. Measuring total S-IgG levels after 3 months do not offer a complete picture of the virus neutralization capacity. And, total S-IgG levels cannot be the sole predictor for adequate immunity post-vaccination and is not sufficient to determine who needs a 3^rd^ vaccine dose.

The authors need to be consistent using the terms SARS-CoV-2 and COVID-19.

The authors use the terms “injections, vaccination, and dose” and “spike IgG titers, values and levels” interchangeably. There needs to be a consistency in the use of specific terms.

**Abstract**

Page2, Line 15: The term full vaccination needs to be properly defined, especially since the authors did not assess the antibody levels after the 3^rd^ vaccine dose. There is not a clear conclusion about the results shown in the manuscript

**Introduction**

Page 4, lines 51-53: The authors make the following statement “Since we found that spike IgG titers two-weeks after the second dose of BNT162b2 vaccine vary widely, we hypothesized that not all subjects should need a third vaccine dose within a fixed time window”. The authors likely need more evidence to substantiate this hypothesis.

**Materials and Methods**

Antibody test

Authors need to describe the Abbott Antibody test in detail. How are the assay cut-off values or S and N-IgG calculated?

Page 6, line 84: clarify the negative cut-off value

Statistical analysis

Table 1: A Student’s t test is not the appropriate test for this data set. The statistics should have been calculated using an ANOVA or non-parametric test with corrections for multiple comparisons.

Page 6, lines 101-103 The authors need to add a proper reference to the study they used to determine the S-IgG cut-off value of 300 AU/ml to stratify the study subjects.

**Results**

All Figures need better labeling throughout and added to the manuscript in a printable resolution.

Page 7, Line 112: Inclusion criteria needs to be described the methods

Line 114, the authors need to describe the details of their previous study in the methods and not in the results section

Figure 1

Figure 1 labeling is confusing. sIgG 1, 2, and 3 usually refers to the subtypes and hence the X-axis labels need to be modified properly to reflect the time points. S-IgG AU/ml data should be log-transformed or presented on a log scale for low-end readability.

Figures 3 and 4

Correlation coefficients need to be included

Discussion

Page 12, line 236: define the terms “excellent and good accuracy”.

Page 13, lines 255-260: the authors need to explain how the current study compares to the study from Israel.

Page 13, line 263: This sentence is confusing and needs to be re-written

Page 14, lines 267-269: There is no sufficient evidence in this study to substantiate this claim

Page 14, line 279: line 286: explain “decay”

Page 15, lines 293-294 term “after vaccination” needs to be defined.

Page 15, lines 313-315: The authors need to explain how they came with the S-IgG cut-ff value of 300AU/ml to stratify the study subjects.
