# Supplementary material for: Temporal changes in spike IgG levels after two doses of BNT162b2 vaccine in Japanese healthcare workers: Do spike IgG levels at 3 months predict levels 6 or 8 months after vaccination?
Source: PLoS One. 2022 Jun 17;17(6):e0263486. doi: 10.1371/journal.pone.0263486 (PMC9205520; doi:10.1371/journal.pone.0263486)
Supplement: Attachment — Submitted filename: Response to Reviewers.docx [file pone.0263486.s005.docx]

We thank the reviewer for his/her thoughtful and constructive comments. In the following itemized response, reviewer comments are in ***bold italic black***. Our responses are in blue, and changes to the revised manuscript are in red.

**To the Reviewer #3:**

***Dear Authors,
Thank you for addressing comments listed in the first round of the review process. I find this new modified version to be greatly improved even though I feel that there are still some minor issues left. Please check my comments and try to provide additional modifications where appropriate or discuss the rationale for opposing.***
Thank you for the positive comments.

**Introduction:
*lines 51-52: “Due to limited vaccine supply, an individualized booster vaccine schedule is mandatory to conserve vaccine doses and to prioritize them to high-risk patients or developing countries.” try replacing “mandatory” with synonyms (i.e., advisably/needed/recommended), since mandatory seems too strong, or please provide explanation if inappropriate.***
We replaced “mandatory” with “recommended”.

Page 4, line 52:

an individualized booster vaccine schedule is recommended to conserve

***in lines 55-57 the Authors explained that the rationale for choosing the 3rd month for measuring the Ab level based on a study [ref 22] that are actually the recommendations applied to patients with cancer. I'm not sure whether this is appropriate since is a particular population there in which (due to the disease and treatment) the Ab dynamics might be different? Also, the following sentence: “We hypothesized that spike IgG level at 3 month is a good time to assess the kinetics of waning immunity because all vaccinees have already reached peak levels of spike IgG.” lacks appropriate reference? Based on what these hypotheses were derived? This needs to be explained.***
We incorporated appropriate references and modified the sentence as follows.
Page 4, Lines 59-61:

We hypothesized that spike IgG level at 3 months is a good time to assess the kinetics of waning immunity because all vaccinees have already reached peak levels of spike IgG [4, 13, 25-27], and some studies found a decline in antibody levels at 3 months [4, 13, 14, 26, 28].

**Methods:**
***The authors provided little satisfactory response to my previous comment regarding the cut-off values: ”Why there was a cut-off at 50 AU/mL? further, a reference would be helpful. RESPONSE: We added references.”
Unfortunately, the provided reference (<https://www.fda.gov/media/146371/download>) also contains little information regarding the cutoff values with and additional remark: “Interpretation of Results -The cutoff is 50.0 AU/mL. As with all analyte determinations, the result should be used in conjunction with information available from clinical evaluation and other diagnostic procedures.” I guess this is still unclear in the literature and without a consensus thus I feel these results should be interpreted with caution.***
Thank you. We incorporated one reference to verify the diagnostic accuracy of the cut-off value (50 AU/mL) in this revised manuscript.

Page 6, line 91:

units/mL (AU/mL) (<https://www.fda.gov/media/146371/download>) [29].

Page 26, lines 519-522

29.	Narasimhan M, Mahimainathan L, Araj E, Clark AE, Markantonis J, Green A, et al. Clinical Evaluation of the Abbott Alinity SARS-CoV-2 Spike-Specific Quantitative IgG and IgM Assays among Infected, Recovered, and Vaccinated Groups. J Clin Microbiol. 2021;59(7):e00388-21. doi: 10.1128/jcm.00388-21 PMID - 33827901.

***Other previous comment: “the authors state that “Subjects were divided into two groups according to age (below 50 years or 50 years and above)”. What is the rationale behind this? Which hypothesis is tested there? This is much unclear, especially because a table with general description of the participants is missing.” The Authors now have made modifications and provided RESPONSE: “Thank you for these thoughtful comments. We just used 50 years as the cut-off as in previous study (PLoS ONE 2021;16:e0257668). We agree that it did not make sense, so we used a new age cut-off here, the median age in this revised manuscript.” Even though it has some sense stratifying data based on the data characteristics, I'm still not sure whether it is appropriate? it’s still unclear which hypothesis was tested here?***
Since age was not a significant covariate for multivariate linear regression analysis (Table 2), we deleted the sentences regarding subgroup analysis using age and in the previous figure 3.

Page 6, lines 105-106:

groups according to ~~median values of age (below 39 years or above 39 years) or~~ gender.

Page 12, lines 205-207

Linear regression analysis was performed separately for ~~age groups stratified by the median age (39 years) (Fig.3) and~~ gender (Fig.3). ~~There were no significant differences in regression slope and intercept between age ≥ 39 years and age < 39 years in any comparisons.~~

Page 12, lines 211-212:

**~~Fig 3.~~** **~~A linear regression analysis between spike IgG levels at 3 and 6 months (panel A), at 8 months (panel B), and at 6 and 8 months (panel C), stratified by age group~~**

Page 19, lines 339-340:

~~and between subjects above and below median age~~.

***Finally, this is still my major concern from the previous round: “line 211: regarding the predictive accuracy - this part is a bit challenging, using data that was used to derive formula for prediction, as a comparison and for the evaluation of its accuracy doesn’t sound quite solid. It seems quite reasonable that in this situation and on the identical population, the prediction would fit as good as it does here but this doesn’t allow for prediction in other population? This is a particular issue since there are several publications using more sophisticated statistical techniques and are still arguing this. Please consult the following reading [doi.org/10.1371/journal.pone.0183250](http://doi.org/10.1371/journal.pone.0183250) and perhaps reconsider presenting this analysis.” the Authors provided RESPONSE: “Thank you for the thoughtful comments. We read the paper that you mentioned, and found that the content was not easy to understand. Anyway, we incorporated VEcv. We think that Lin’s concordance correlation coefficient (CCC) is another metric to quantify accuracy; thus, we also included Lin’s CCC in this revised manuscript.” Additionally, I agree with the comment 5 from the Reviewer #1 that said “a comparison of the equations obtained applying the same method described in this study to other published cohorts will be useful to further support the accuracy of the predictions for healthy individuals of different ethnicities.” In fact, the S1 Fig shows good correlation between predicted and measured values, which is expected when using the same dataset, as I previously explained. I feel this is another limitation of the study. The problem is not comparison to other ethnicity as listed in the lines 358-360 but the methodology (data) used for accuracy evaluation.***
Thank you for these important comments. We agree with your comments, and described these points in the study limitation.

Page 21, lines 382-386:

Third, although we generated simple equations, a good correlation between predicted and measured values was expected because we used the same dataset. A comparison of the equations obtained applying the same method described in this study to other cohorts will be useful to further support the accuracy of the predictions for healthy individuals of different ethnicities.

**Results:**
***Table 1: please provide appropriate labeling of the Variables i.e. Age (years), median (IQR); Male, n (%), etc. What represents the Part-time occupation? it’s unclear, it doesn’t seem a position on which they work? should be explained or modified. I don’t feel “Nucleocapsid IgG at 3/6/8 months” values should be listed here in Table 1 and are a bit misleading since these are not characteristics of the participants but something that was measured in this study and also only 3 participants were positive on this test so median of 0.02 doesn’t tell much since many had value of 0?***

Thank you for these good suggestions. We incorporated them in table 1. “Part-time” means part-time worker, but it changed it to “Others”. We omitted nucleocapsid IgG values in table 1 as suggested.

***Correlation coefficients (r) should be accompanied by corresponding p-values (lines 164, 165, etc.)***
We incorporated p-values.

Page 9, lines 168-169:

months (r=0.86, p<0.001) and between 3 and 8 months (r = 0.82, p<0.001). The correlation was almost perfect between 6 and 8 months (r = 0.98, p<0.001).

***As per propositions of PLOS One, tables should be placed right after being first mentioned in the text (Table2).***
We did.

***I find information from the nomograms presented in Table 4 (and 5) to be very useful. But, how was the formula used for the Predicted elapsed month = 37.5 * (1 – (2.71 / log-transformed spike IgG at 3 month)) derived? There is no explanation on this?***
We added these sentences to describe how to obtain this equation in the foot note of Table 5.

Page 15, lines 266-270:

Values of the second raw of the table was calculated as follows.

From two formulae (log spike IgG at 6 months = 0.92 X log spike IgG at 3 months – 0.23, log spike IgG at 8 months = 0.86 X log spike IgG at 3 months – 0.18),

We supposed that the coefficient declined linearly according to month (M) (0.08/3 = 0.027, 0.14/5 = 0.028).

Thus, log spike IgG of 300 = (1 – 0.027 M) x log spike IgG at 3 months – 0.23.

Predicted elapsed month (M) = 37 x (1 – (2.71 / log spike IgG at 3 month))

**Discussion:
*I would be more satisfied if the authors used some of the studies listed in lines 285-287 “Several studies have measured short- [28, 29] and long-term changes [4-14] in spike IgG and neutralizing antibody levels up to 9 months after two doses of BNT162b2, and all studies have revealed that antibody levels drop over time.” to make more vivid discussion, i.e., to compare their results with those obtained by previous studies (since there are many available nowadays).***

Thank you for the good suggestions. We made Table 6 in which we compared spike IgG levels at 3 and 6 months after two-dose BNT162b2 vaccination and their percent reductions, and discussed the observed results.

Page 17-19, lines 311-325:

Table 6 summarizes reported spike IgG levels at 3 and 6 months after two doses of the BNT162b2 vaccine.

**Table 6: Reported spike IgG levels at 3 months and 6 months after two doses of BNT162b2 vaccine**

| Author | Study type | Subjects | Country | Spike IgG at 3M | Spike IgG at 6M | % reduction |
| --- | --- | --- | --- | --- | --- | --- |
| Bayart | longitudinal | HCWs | Belgium | 6,050 AU/mL (n = 158) | 949 AU/mL (n= 158) | 68% |
| Naaber | longitudinal | HVs | Estonia | 5,226 AU/mL (n = 122) | 1,383 AU/mL (n = 122) | 74% |
| Rode | longitudinal | HCWs | Croatia | 2,977 AU/mL (n = 405*) | 966 AU/mL (n = 337*) | 68% |
| Guiomar | cross-sectional | HCWs | Portugal | 6,812 AU/mL (n =32) | 1,070 AU/mL (n= 72) | 86% |
| Israel | cross-sectional | PB | Israel | 2,383 AU/mL (n = 200) | 765 AU/mL (n = 440) | 68% |
| Kertes | cross-sectional | PB | Israel | 2,706 AU/mL (n = 827) | 1,411 AU/mL (n = 1,820)** | 48% |
| Our study | longitudinal | HCWs | Japan | 2,882 AU/mL (n= 251) | 875 AU/mL (n = 250) | 70% |

Spike IgG levels are presented as mean or median.

HCWs, health care workers; HV, healthy volunteers; n, number; M, month; PB, population based.

*: exact number not available

**: ≥150 days

IgG levels at 3 and 6 months varied widely among studies. The percent reduction of spike IgG levels from 3 months to 6 months ranged from 48% to 86%. However, it is interesting to note that the percent reduction of spike IgG levels in our study (70%) was quite similar to the corresponding value observed in three longitudinal studies (68%, 74%, 68%), which suggests that the decay kinetics of spike IgG after two-dose BNT162b2 vaccination may be relatively constant.

***Also, the results seem quite valuable but in the discussion I miss the answer on the major question “what might be the utility of these findings” and stronger statement on why these results are valuable for practice?
line 339 seems a bit misleading, these are more Public health implication rather than Clinical implications? this section should answer the question: What would these findings add if being implemented in practice?***

We modified the sentences in Clinical implication to try to answer these questions.

Page 20-21, lines 364-376:

According to the table, spike IgG levels of 900 AU/mL represent approximately the minimum amount of spike IgG necessary at 3 months to maintain spike IgG of ≥ 300 AU/mL at 6 months. The table also provides minimal spike IgG levels at 3 months required to maintain spike IgG levels of 500, 750, 1,000, 1,500 and 3,000 AU/mL at 6 or 8 months. This table provides predictive spike IgG levels at 6 months in individual subjects, for whom we have spike IgG levels at 3 months after two-doses of BNT162b2 vaccine. Since spike IgG levels at 3 months vary widely among subjects, this information is quite useful for individualized third-dose vaccine schedules. Another nomogram (Table 5) shows at which month after vaccination one expects to reach the pre-defined spike IgG cut-off level (300 AU/mL) based on levels at 3 months. This nomogram provides information when spike IgG levels become < 300 AU/mL in each subject who measured spike IgG levels at 3 months; thus, it supports individual scheduling for a third dose.

We added six references to this revised manuscript. (#13, 14, 25, 27, 28, and 29).
